# Stigmatization experiences of healthcare workers in the context of the COVID-19 pandemic: a scoping review

**DOI:** 10.1186/s12913-024-11300-9

**Published:** 2024-07-17

**Authors:** Reza Negarandeh, Mehraban Shahmari, Leily Zare

**Affiliations:** 1 Nursing and Midwifery Care Research Center, School of Nursing and Midwifery, Tehran University of Medical Sciences, Tehran, Iran; 2 Department of Medical-Surgical, School of Nursing and Midwifery, Ardabil University of Medical Sciences, Ardabil, Iran; 3 Department of Medical Surgical, School of Nursing and Midwifery, Tehran University of Medical Sciences, Tehran, Iran

**Keywords:** Stigma, Health Workers, COVID-19

## Abstract

**Background:**

During the COVID-19 pandemic, healthcare workers face the stigmatization of those caring for COVID-19 patients, creating a significant social problem. Therefore, this study investigated the stigmatization of healthcare workers in the context of the COVID-19 pandemic.

**Methods:**

In this scoping review study, searches were conducted from December 2019 to August 2023 in Persian and English using various databases and search engines including PubMed (Medline), Embase, Scopus, ISI Web of Science, ProQuest, Science Direct, Springer, Cochrane Library, Google Scholar, and national databases. The study used English keywords such as Social Stigma, Health Personnel, Healthcare Worker, Medical Staff, Medical Personal, Physicians, doctors, Nurses, nursing staff, COVID-19, and coronavirus disease 2019, and their Persian equivalents, and their Persian equivalents to explore healthcare workers’ experiences of COVID-19-related stigma.

**Results:**

From a total of 12,200 search results, 77 eligible studies were included in this study. stigmatization of healthcare workers caring for COVID-19 patients was evident from the literature because of fear, misinformation, and negative self-image. Manifestations were violence and deprivation of social rights, resulting in adverse biopsychosocial, occupational, and economic consequences. This condition can affect negatively health staff themselves, their families, and society as well. Anti-stigmatization measures include informing society about the realities faced by healthcare workers, presenting an accurate and empathetic image of health workers, providing psychosocial support to health workers, and encouraging them to turn to spirituality as a coping mechanism. There are notable research gaps in comprehending the phenomenon, exploring its variations across diverse healthcare roles and cultural contexts, examining its long-term effects, and monitoring shifts in stigma perceptions over time.

**Conclusion:**

The COVID-19 pandemic has resulted in the stigmatization of healthcare workers, causing mistreatment and rights violations. This stigma persists even post-pandemic, posing a psychological dilemma for caregivers. Addressing this requires comprehensive strategies, including tailored stigma prevention programs and research to understand its psychological impact.

## Background

The COVID-19 pandemic, recognized as a global traumatic event, has profoundly disrupted various aspects of daily life worldwide [1] and has put unprecedented and significant pressure on the healthcare system and its healthcare workers (HCWs) [2]. Despite the important role of HCWs in the fight against infectious diseases [3], this pandemic has brought hard times for them worldwide. Being on the frontline of fighting this pandemic has had many direct and indirect negative effects on them, from physical and psychological injuries to professional and social issues [4]. So much so that these conditions began to be seen as a social problem [5]. One challenge faced when working with COVID-19 patients is the stigma HCWs may encounter due to being seen as a serious threat, posing a significant hurdle in the healthcare field [6]. In health, stigma refers to the labeling and discrimination of people based on a particular disease [7]. According to the WHO [8], social stigma in healthcare is a negative association between a group of people and a particular disease [8]. Even people who do not suffer from this disease but have other common characteristics, such as family members or HCWs responsible for their care, can suffer from this stigma [9]. Stigmatization is the main cause of discrimination and disadvantage, leading to a violation of human rights [10]. The stigma associated with COVID-19 had consequences such as increased fatigue, burnout, and lower satisfaction, which affected the well-being of HCWs [11]. In addition, perceived stigma impaired feelings of self-efficacy and increased psychological and psychophysical problems [12]. According to the available literature, stigma is a complex phenomenon that is experienced differently depending on the type of illness and the social conditions of those affected [13]. On the other hand, the effects and consequences of stigma experienced by stigmatized people can persist in the long term, even after the end of quarantine and the containment of the epidemic [11].

The history of previous epidemics, including SARS and MERS, has shown that HCWs were usually considered carriers of the virus and were transmitted by others because of this status [14, 15]. This situation not only had a negative impact on their mental health [16–18], but also affected their desire to remain in the profession [19]. Additionally, during the COVID-19 pandemic, some studies, including systematic reviews, have addressed the stigmatization of HCWs [4, 20]. However, due to the ongoing pandemic, a re-examination of changes in healthcare professionals’ experiences of stigmatization and its impact on their personal and professional lives is needed [21, 22]. Conducting review studies makes the role of stigma in changing work relationships, mental health, and professional experiences clearer and offers effective solutions to support health professionals in critical situations [22–24]. In addition to providing a deeper understanding of the factors that influence stigma, its manifestations, and its social, psychological, and occupational effects, this review study aims to fill the gap by providing updated insights and further clarifying the role of stigma in altering work relationships, mental health, and professional experiences and offers individual and organizational strategies to address these issues and support HCWs.

## Method

### Study plan

This review used Arksey and O’Malley’s [25] six-level framework to review comprehensive texts [25]. To examine the number, scope and nature of available studies on stigma related to COVID-19 in HCWs. The results are reported according to the guidelines of the PRISMA Extended Program for Scoping Review (PRISMA-ScR) [26]. This approach consists of six steps: 1) defining the research question, 2) identifying related studies, 3) criteria for selecting studies, 4) capturing and classifying key findings (e.g. study location, intervention, comparison, study population, study objectives, outcomes, measures and conclusions, etc.), 5) summarizing and reporting results, and 6) consulting stakeholders (optional). The purpose of a scoping review is a detailed examination of the texts in a specific area without assessing the quality of the studies. Therefore, qualitative assessments are often not performed and studies are not critiqued [25]. Therefore, no qualitative assessment of the articles was performed in the present study. Each of the steps carried out in this study is explained below.

### Determining the research question

The present scoping review was guided by the following questions: a) Has the COVID-19 pandemic triggered a recognizable manifestation of stigmatization of HCWs? b) What symptomatic consequences have been observed in HCWs as a result of COVID-19-related stigmatization? c) What are the tangible and intangible effects of COVID-19-related stigma on HCWs? d) What proactive measures could be taken to reduce the incidence and impact of COVID-19-related stigma among HCWs? e) What research gaps exist in the study of COVID-19-related stigma among HCWs that require further investigation?

### Identification of related studies

The studies utilized in this research were sourced from a comprehensive search across various databases and search engines, including PubMed (Medline), Embase, Scopus, ISI Web of Science, ProQuest, Science Direct, Springer, Cochrane Library, Google Scholar, and National databases. This extensive search strategy involved English keywords such as Social Stigma, Health Personnel, Healthcare Worker, Medical Staff, Medical Personal, Physicians, doctors, Nurses, nursing staff, COVID-19, and coronavirus disease 2019, and their Persian equivalents, combined using Boolean operators AND, OR, * to ensure inclusivity and exhaustiveness.

One example of the search strategy using MeSH terms in PubMed is listed in Table 1.

**Table 1:** Search strategy

PubMed (Medline)	((((((“Health Personnel”[Mesh])) OR “Medical Staff”[Mesh]) OR “Physicians”[Mesh]) OR “Nurses”[Mesh]) AND “Social Stigma”[Mesh]) AND “COVID-19”[Mesh]

### Study selection criteria

The following inclusion and exclusion criteria were applied in the review of titles/abstracts and full text to determine the final number of studies:

- All English and Persian studies with available full texts published between December 2019 and August 2023 related to the stigmatization of HCWs during the COVID-19 pandemic.
- HCWs including nurses, doctors, healthcare professionals, paramedics, and technicians.
- Studies relating to previous epidemics were excluded.

After a comprehensive search, the studies were reviewed and selected in a two-stage process. The studies were screened by two researchers (M. Sh. and L. Z.). In the first stage, the titles and abstracts were reviewed independently by two researchers to determine the eligibility of the studies. The studies were classified as relevant, possibly relevant, or not relevant. In the second stage, the potentially relevant studies were reviewed independently by the researchers to determine final eligibility. Research results were then compared and duplicates were removed using EndNote-X9 software. Researchers were present at both stages of the meeting to share opinions and reach consensus, and a third party (R.N.) was consulted when necessary. The sources of all studies eligible for further review that were not provided in the electronic database search, the review, and the search strategy were documented and stored in each database, and the search results were stored in the EndNote-X9 resource management tool. The PRISMA-ScR diagram shows the process of searching and selecting texts.

### Registration and classification of key results

After selecting the texts, the data were extracted and recorded in tables in Microsoft Word 2023. The main areas of focus in the data included authors, date of publication, country, type of texts, sample characteristics, and key findings (Table 2).

**Table 2:** Characteristics of included studies

No	Author(year)	country	Literature type	Sample characteristics	Key findings
1	Nashwan et al. [30]	Multi countries	Cross-sectional	1726: Physician (*n* = 405), Nurse (*n* = 932), Pharmacist (*n* = 250) Allied health (*n* = 139)	Significant increase in COVID-related stigma towards HCWs **Consequences:** fear of being infected with COVID-19 **Preventive measures:** strengthened public awareness of COVID-19, the provision of a safe workplace equipped
2	Abdel Wahed et al. [31]	Egypt	Descriptive cross-sectional	407: Physician (*n* = 127), Nurse (*n* = 102), Pharmacist (*n* = 36), Technician (*n* = 36), Employee (*n* = 79), Housekeepers (*n* = 37)	perception of stigma related to COVID-19 equals to 66.3% **Consequences:** fear of COVID-19 infection **Preventive measures:** proper education, clear announcing of healthcare policies, launching stigma reduction programs
3	Verma et al. [104]	Korea	Cross-sectional	public health doctors (*n* = 350)	Perceived stigma from family and friends (worries for possible transmission of infection through public health doctors at frontline) and rejection from the neighborhood **Consequences:** Predicted anxiety and depressive mood **Preventive measures:** greater psychosocial support from family, friends, and supervisors, better cooperation between colleagues at the workplace, proper educational training on COVID-19 for the healthcare professionals, dissemination of clear information to the general population
4	Park et al. [69]	Korea	Cross-Sectional Study	1,003: Doctor (*n* = 71), Nurse (*n* = 648), Medical technician (*n* = 98), Administrative and secretary officers (*n* = 74), Pharmacy staff (*n* = 12) Cafeteria workers (*n* = 18), Others = 82	Experienced social rejection or had other negative experiences **Consequences:** depression and anxiety **Preventive measures:** Need for appropriate psychological intervention measures to ensure healthy work environments for HCWs
5	Mostafa [62]	Eygpt	Cross-Sectional Study	Physicians (*n* = 509)	The mean overall COVID-19-related stigma score was 40.6 ± 8.0. The mean scores for the subscales were: personalized stigma 26.0 ± 5.7, disclosure concerns 9.3 ± 2.2, negative self-image 6.9 ± 1.6, and concern with public attitude 24.4 ± 4.9 **Consequences:** stay away from their families, feeling guilty, hiding a positive test result **Preventive measures:** Public health education and raising community and media awareness about the importance of public support for HCWs, need for specific research and targeted interventions particularly addressing COVID-19-related stigmatization among HCWs
6	Makino et al. [53]	-	Commentary	-	The experience of stigma by HCWs and their families **Consequences:** psychological pressure, even suicide **Preventive measures:** plan to promote mental health
7	Chu et al. [58]	USA	Cross-sectional survey	402: Medical Providers (*n* = 184), Registered Nurses (*n* = 218)	Perception of social stigma
8	Rahmani [59]	Iran	Phenomenological Study	Nurses (*n* = 12)	Stigma in the form of self-isolation, social isolation and rejection by friends, family and neighbors **Consequences:** Experiencing mental stress in the form of anger and rage, loneliness and humiliation, depression **Preventive measures:** Relying on God and not depending on others and increasing the level of knowledge, the effective role of the media in changing the attitude of the society towards nurses by broadcasting their dedication
9	Bagheri et al. [56]	-	Letter to editor	-	Social stigma and self-stigma and the experience of stigma by their family **Consequences:** Limiting social communication and being more present at home and at work **Preventive measures:** It is necessary to take measures to socially motivate the medical staff of hospitals by the Ministry of Education and Health
10	Simeone et al. [55]	Italy	Phenomenological Study	Nurses (*n* = 19)	Stigma in the working environment **Consequences:** Stigma can be more dangerous than the disease, and a major obstacle to appropriate medical and mental health interventions **Preventive measures:** Need to design and implement specific educational, psychological, and organizational programs
11	Jeleff et al. [105]	Austria	qualitative study	Medical doctors(*n* = 13), qualified nursing staff (*n* = 11), nurse (*n* = 2) assistants (*n *= 2), physiotherapists (*n* = 2) and technical/cleaning staff (*n* = 2)	Stigma and avoidance behavior in private life and by colleagues, self-stigmatization or avoidance behavior such as sleeping in separate bedrooms or not kissing their partner **Preventive measures:** Need to care for the mental health of HCWs
12	Adhikari et al. [106]	Nepal	Cross-Sectional Survey	Other than the doctor (*n* = 94) Doctor (*n* = 119)	More than half of HCWs faced some form of stigma in society due to COVID-19 **Consequences:** Depression and anxiety
13	Menon et al. [ 71]	India	Cross-Sectional Survey	Doctors(*n* = 173), Auxiliary nurse / paramedical staff(*n* = 103), Nurses(*n* = 190) Laboratory staff/ Supporting staff (*n* = 142), House-keeping /sanitation(*n* = 89), Ambulance driver/staff/ward boys/Guards(*n* = 162), ASHA/UHW/USHA(*n* = 108)	Societal stigma against hospital workers **Preventive measures:** Implementing the most stringent preventive measures, reducing the anxiety/stigma associated with COVID-19 transmission, and providing adequate psychological and social support will significantly lower occupational stress among healthcare professionals
14	Zolnikov et al. [33]	Multi countries	Phenomenological Study	31 physicians, nurses, paramedics, police officers, firefighters, etc	Feelings of isolation, lack of support and understanding by family or friends, decreased or forced removal in immediate social interaction (e.g., within family and friend circles), sentiments of being infected or dirty, increased feelings of sadness and anxiety, and reluctance to ask for help or get treatment (e.g., self-approval of being isolated)
15	Ramaci et al. [11]	Italy	Cross-Sectional Survey	nurses (*n* = 67), doctors (*n* = 206)	Stigma positively impacts fatigue and burnout, and negatively impacts satisfaction among HCWs **Preventive measures:** Providing information and increasing awareness
16	George et al. [63]	India	A mixed method study	Doctors(*n* = 20), Nurses (*n* = 14), Field staff (*n* = 14), Allied health professionals (*n* = 10), Others (*n* = 6)	Experienced emotions of fear, anxiety and stigma during the pandemic **Preventive measures:** Peer support, distancing, information seeking, response efficacy, self-efficacy, existential goal pursuit, value adherence and religious coping
17	Nyumirah [64]	-	Literature Review	8 articles	Feelings of anxiety occur because of social stigma related to this pandemic condition **Preventive measures:** providing mental and psychosocial health support for HCWs to overcome the impact of psychological problems that occur and increase immunity during the COVID-19 pandemic
18	Handayani et al. [20]	-	systematic review	10 articles	Perception of stigma **Consequences:** Causing stress
19	Vani et al. [75]	-	Invited Perspective/Commentary	-	The close proximity of working with infected individuals has led to significant stigmatization of the HCWs in society **Consequences:** fatigue and negative outcomes such as burnout, and inversely impacts work satisfaction among HCWs
20	Abuhammad et al. [48]	Jordan	Cross-Sectional Survey	People (*n* = 777)	Many people show a high stigma toward HCPs during the COVID-19 pandemic
21	Timothy et al. [38]	Multi country	Cross-Sectional Survey	HCWs (*n* = 837), non- HCWs (*n* = 6574)	Experience of harassment, bullying, and hurt and powerful stigma by HCWs and their family **Preventive measures:** Psychological support, Increased awareness and information
22	Alajmi et al. [50]	Saudi Arabia	Cross-Sectional Survey	226 HCWs	The data extracted three factors: communication impairment, social avoidance, stigma, and personal deprivation and distress, rated as severe, moderate, and moderate, respectively. Discontinued workgroups are more affected by communication impairments, social avoidance, and stigma, and less emotional and personal deprivation
23	Argyriadis et al. [44]	Greece	An ethnographic approach	160 interviews	Health professionals faced discriminating behaviors and stigma from their families, social environment, and other health professionals
24	Jain et al. [107]	India	Cross-Sectional Survey	120 frontline HCWs	Out of 120 frontline HCWs participated in the study, 68 (56.6%) reported severe levels of COVID-19-related stigma. Severity of stigma was associated with age, male gender, designation, education, and marital status of HCW
25	Trusty et al. [51]	USA	survey online	112 primary care providers	Public care personals perceived public stigma (e.g., beliefs that seeking psychotherapy is shameful) becomes internalized as self-stigma (i.e., beliefs that one’s self-esteem would be reduced by seeking psychotherapy). In turn, self-stigma may lead to negative attitudes toward seeking psychotherapy, such as beliefs that it will be unhelpful or limit professional opportunities. Finally, negative attitudes may then impede intentions to seek psychotherapy when needed
26	Saptarini et al. [34]	Jakarta	cross-sectional online study	277	The negative self-image dimension is the dimension most felt by HCWs. More than half of HCWs agreed that during the COVID-19 pandemic, they put their families at risk because of their status as HCWs. The stigma of HCWs who work in hospitals is higher than that of non-hospital HCWs, such as health centers, clinics, and laboratories
27	Kwaghe et al. [29]	Nigeria	Colaizzi’s phenomenological	20	Stigmatization (stigmatized by colleagues, family, friends, or their residential communities, reasons for stigmatization which was fear of infection, limited knowledge of the virus and working at the isolation center and the effect of stigma)
28	Schubert et al. [4]	-	Systematic Review with Meta-Analysis	46 articles	Generally, all included studies indicate that stigmatization occurs as a result of work-related COVID-19 exposure
29	Khalid et al. [45]	Pakistan	Cross-Sectional Survey	134 HCWs	51.5% of HCWs felt stigmatized due to working during the COVID-19 pandemic by the people surrounding them including their families and communities
30	Osman et al. [108]	Eygpt	Cross-Sectional Survey	565 HCPs	Considerable levels of worry and stigma perceptions were detected among Egyptian HCPs during the COVID-19 outbreak
31	Trejos-Herrera et al. [109]	-	VIEWPOINTS		HCWs experienced different types of stigmas
32	Ampon-Wireko, Zhou et al. [78]	Ghana	A descriptive cross-sectional study	820	COVID-19 stigmatization among frontline HCWs directly affects their job performance
33	Gratton [27]	Canada	Thesis		Social stigma is a common experience for frontline workers during outbreaks and is driven by people’s fear of contracting the illness
34	Gualano, Sinigaglia et al. [52]	-	A Systematic Review		Social stigma from community is one of the risk factors for burnout in ICU/ED HCWs
35	Woga et al. [83]	Indonesia	Cross-Sectional Survey	1,697 nurses	The stigmatization variable COVID-19 has no effect on the performance of nurses in this study
36	Wickramasinghe et al. [47]	Sri Lanka	Secondary analysis	924	Personnel working in health, security, and other essential services and their family members experienced stigma and discrimination from the wider society, neighbors, or media
37	Winugroho et al. [79]	Indonesia	Cross-Sectional Survey	63	The length of quarantine and stigmatization simultaneously influence the resilience of COVID-19 survivor nurses
38	Wahlster et al. [110]	Multi country	Cross-Sectional Survey	2700	One of the most common concerns included experiencing social stigma in their communities
39	Jahangasht- [111]	-	View point	Patients with COVID-19 disease and its carriers	Social stigma causes symptoms such as fear, anxiety and depression both in ordinary people and in medical and therapeutic staff, and this weakens the immune system of these people. Stigma can weaken social cohesion and increase the possibility of social isolation. Excessive anxiety makes people accept rumors more and fuel rumors to reduce their fear and worry. This may lead to loss of social status due to perceived association with a particular disease. Sometimes this stigma causes a person to suffer isolation and deprivation of social and civil rights and even deprivation of support from his family Actions: The first step in de-stigmatizing the COVID-19 disease is social care (social care is usually the responsibility of institutions that provide social services). The next step is social empathy
40	Shafiei- [80]	-	Letter to editor	health personnel	It causes a person to suffer isolation and deprivation of social and civil rights and even deprivation of the support of his family.” Actions: According to WHO, the society should not label the people who take care of these people, wear masks correctly and observe social distance, hold training sessions and provide adequate and up-to-date information to the society During the outbreak of COVID-19, even the families of hospital staff experienced this social stigma and many people cut off and limited their contact with them
41	Schubert et al. [4]	-	Systematic Review	46 articles	Based on the investigations, most studies reported a high prevalence of social stigma to the medical staff. On the other hand, the social stigma against nurses and doctors caused an increase in anxiety and depression in people, which resulted in a decrease in the mental health of medical staff during the COVID-19 pandemic
42	Fathi Ashtiani- [112]	-	Systematic Review	A total of 36,602 people from the general public, athletes, students, medical staff of hospitals and vulnerable groups such as the elderly and people with chronic diseases	The COVID-19 has led to the creation of social stigma in the medical staff of hospitals. The social stigma related to COVID can have bad effects on the performance and life of the medical staff
43	Cuong Do Duy [113]	-	Letters to the Editor	response pattern, and included questions about foreign people, patients with COVID-19, and	The median of Stigma Scale total score was 11 (interquartile range = 6–15; min.–max. = 0–24). Three dimensions were reconstructed from factor analysis: (i) Negative Self-image, (ii) Disclosure Concerns and Personalized Stigma, and (iii) Concerns About Public Attitudes. The success of outbreak containment in Vietnam has been due to the government’s early and constantly aggressive approach as well as its traditional and modern mass media campaign to improve the awareness of all citizens. but this may have inadvertently increased the likelihood of stigmatization of people after quarantine regardless of their infection status. In frontline HCW, the negative impacts could be more serious as they are receiving greater attention in the press and mass media
44	Maryam Vizheh- [114]	-	– An integrative literature review	HCWs	‘Health-related stigma’ is typically known as social rejection or exclusion of individuals and populations suffering from specific health problems stigmatization can considerably increase psychosomatic distress and disturbance. HCWs and volunteers working in the field may also become stigmatized, leading to higher rates of distress, stress, and burnout. Finally, when people avoid groups or geographic areas related to infectious diseases, this can pose significant economic losses
45	Timothy D Dye-[115]	USA	nested mixed method	“7411 people from 173 countries who were aged 18 years or over in four languages (English, Spanish, French, Italian) through	After controlling for a range of confounding factors, HCWs are significantly more likely to experience COVID-19-related stigma and bullying, often in the intersectional context of racism, violence and police involvement in community settings
46	Mariem Turki, [116]	Tunisia	cross-sectional web-based survey	250 Tunisian HCWs	HCWs perceived stigma in professional, societal and familial domains. Participants sometimes to often experienced stigma in their relationships with friends (22%), neighbors (27.2%), parents (22,4%), and in social activities (30.8%). This stigma was perceived mainly through avoidance (68.4%), and rarely through verbal (6%) or physical aggression (1.2%)
47	Ankur Sachdeva, [117]	India	a hospital-based cross-sectional study	150 HCWs involved in COVID-19 care	Stigma was significantly reported in most HCWs, especially with concerns regarding public attitude and disclosure of their work profile. This may lead to long lasting psychosocial consequences which may affect more severely than the infection itself
48	Sanjeet Bagcchi, [118]	-	report	150 HCWs involved in COVID-19 care	Stigma associated with COVID-19 poses a serious threat to the lives of HCWs and there has been more than 200 incidents of COVID-19 related attacks on HCWs and health facilities during the ongoing pandemic. They experience avoidance by their family or community owing to stigma or fear, like denied access to public transport and were subjected to physical assaults, insulted in the street, and evicted from rented apartments. HCWs have to face substantial stigma during the pandemic as a result of the fear. facing harassment at public places because they have been perceived as at higher risk of transmission
49	Ramdas Ransing, [9]	-	letter		In the majority of represented countries, COVID-19 stigma, as a global phenomenon, was associated with similar drivers, (e.g., fear associated with the infection or the quarantine), beliefs (supra-natural or religious), and blame to self or others for contracting the disease, as well as guilt and shame. HCWs deployed in COVID-19 services have experienced discrimination such as the refusal of housing, verbal abuse or gossip, and social devaluation. Also, their family members or friends are experiencing ‘secondary’ or ‘associative’ stigma. In societies decreased acceptability of HCWs in their communities, and overall decreased resilience (i.e., power to challenge stigma) may jeopardize their health and wellbeing
50	Steven Taylor, [39]	Multi country	online survey	Non-HCW adults from the United States and Canada (*N* = 3551)	Over a forth respondents believed that HCWs should have severe restrictions placed on their freedoms, such as being isolated from their communities and families. Over 1/3 of respondents avoided HCWs for fear of infection. Participation in altruistic support of HCWs (i.e., evening clapping and cheering) was unrelated to stigmatizing attitudes. Demographic variables had small or trivial correlations with HCWs stigmatization. People who stigmatized HCWs also tended to avoid other people, avoid drug stores and supermarkets, and avoid leaving their homes
51	Julian Sheather, [119]	-	Report		Public fear of the virus is morphing into stigmatization of health professionals. Punched in the face in Chicago, doused with bleach in the Philippines, stoned by mobs in India, HCWs, already under enormous strain, are increasingly becoming targets. as of 28 April, the Mexican Ministry of Interior had documented at least 47 acts of aggression against HCWs. There is also a separate harrowing report of a nurse being drenched with chlorine when walking home. The report details health professionals being evicted from homes for fear of infection, refused access to public transport, uniformed Nigerian nurses being denied access to supermarkets, and ambulance staff being assaulted by mobs in Russia
52	Rakesh Singh, [40]	-	Letter to the Editor		The healthcare providers are being labelled, set apart and are facing loss of status and discrimination because of stigma attached with COVID-19. They are too facing mental health challenges. The psychological problems in turn may alter their attention and decisioning capability which is not only limited to affect their mental wellbeing but can also affect in managing the ongoing crisis
53	Shiu, [72]	Taiwan	a web-based, structured survey from March 12th to 29th, 2020 to collect cross-sectional, self-reported data	Of the 1421 consented respondents, 357 identified as physicians while 1064 identified as nurses	Participants reported some levels of stigma and Burnout symptoms were positively correlated with COVID-19 stigma. The interaction between stigma and profession (Stigma Nurses) but no other interaction terms reached the significance level, suggesting that the slope for nurses was flatter than the slope for physicians
54	Sandeep Grover, [10]	-	Letter to the Editor	HCW	information guides in general advise that people should not stigmatize people undergoing quarantine, those with travel history, those who are diagnosed with COVID-19, and those who have recovered from the COVID-19 infection. However, it is still rampantly prevalent. These cases highlight the fact that even the HCWs are behaving the way, as others in the general population, who are less knowledgeable about the mode of transmission
55	Dickson Adom, [81]	Ghana	phenomenology	28 participants who have tested positive but have recovered, suspected COVID-19 persons quarantined in isolation centers, relatives of COVID-19 patients, Ghanaian returnees, and foreign nationals from COVID-19 hotspot countries, frontline HCWs, clinical and social psychologists, and mental health officers	The findings revealed that COVID-19 victims have faced various forms of stigma such as stereotyping, social exclusion, mockery, finger-pointing, and insults
56	Nalakath A. Uvais, [41]	-	Letter to the Editor	335 respondents 55.2% of the dialysis technicians and 44.8% were dialysis nurses;	The mean stigma score was 25.33 (SD = 8.12); indicating high levels of perceived stigma. Our study showed that 54.6% of the dialysis staff perceived significant stigma associated with their job and 36.1% of them significant stress
57	Michael J. Van Wert, LCSW-C, MPH, [120]	USA	Cross-sectional survey in an urban medical center (September–November 2020) in Baltimore, MD, in the United States	605 HCWs (physicians, nurse practitioners, nurses, physician assistants, patient care technicians, respiratory therapists, social workers, mental health therapists, and case managers)	72.4% of HCWs reported high health fear, 50.1% high job stressors, 33.6% high perceived social stigma and avoidance, and 33.6% high workplace safety concern. HCWs who reported high (relative to low) perceived social stigma and avoidance had a higher likelihood of sleep disturbance, PTSD symptoms, and high burnout
58	Paula Franklin, [82]	-	Scope REVIEW	220 articles	HCWs were stigmatized in their communities as virus carriers, they report fear of stigma or discrimination in their neighborhood and are often afraid to go home after work, while also stigma leads to even eviction from accommodation and physical assaults. HCWs are often the targets of intersectional processes of stigmatization across their professional roles, ethnicity, gender, and race. With nurses, women, Black, Asian, and minority ethnic (BAME) HCWs and all those combining these characteristics being more severely affected
59	Jonathan Fan, [42]	-	umbrella review of reviews	review articles published in MEDLINE, PsycINFO and Embase between 2000 and 2020	HCWs experienced considerable stigma during quarantine. Social stigma and rejection associated with working in a high-risk environment seemed to affect workers irrespective of their occupational role. Common coping mechanisms to withstand stigmatization included support from family and peers and seeing their efforts translate to patients getting better
60	Je-Yeon Yun, [121]	Korea	cross-sectional online survey	350 public health doctors with experiences of work at COVID-19 frontline participated	perceived stigma from family and friends and rejection from neighborhood predicted anxiety and depressive mood of PHDs, respectively
61	Birgül Cerit, [122]	Turkey	phenomenology	nine nurses who work in a COVID-19 clinic of a pandemic hospital	stigma is one of the emotions experienced by nurses when they worked in the COVID-19 clinic. Over time, as they gained more experience, anxiety and fear were replaced by happiness and confidence as well as greater feelings of stigmatization
62	Goodluck Nchasi, [87]	-	commentary paper	HCWs in Africa	Social stigmatization and loss of trust in society are other under looked aspects of the pandemic that take a heavy toll on the HCWs
63	Ashraf karbasi, [123]	Iran	cross‑sectional	237 participants including HCWs and their nuclear family members	HCWs and their nuclear family members who are possible carriers of COVID‑19 suffer from severe stigma
64	Belice, [57]	Turkey	cross‑sectional	136 individuals (40 male and 96 female) participated in the study	The stigmatization rate toward healthcare providers was found to be significantly high, and the stigmatization level was found to increase significantly with increasing age. 108 participants (79.4%) were found to have stigma levels at the pathological margin
65	Salman Alsaqri, [124]	Saudi Arabia	descriptive qualitative study	9 participants of frontline nurses from various hospitals in the City of Hail	The participants mostly encountered social stigma when they were asymptomatic and had social contact after their isolation and began their hospital work. The nurses diagnosed positively with COVID-19 felt stigmatized in their workstation and the community during and after complete recovery and undertaking the mandatory quarantine period. researchers expected nurses to be feeling stigmatized only outside of the hospital setting. However, it was discovered in this study that social stigma could be experienced even at the hospital where, supposedly, the place of healthy environments, healthy habits, and healthy human interactions
66	Roelen, [43]	-	Review	-	Driving Factors of COVID-19 Related Stigma were lack of information and misconceptions, fear of contagion, targeted policies. four main fault lines were identified: 1) poverty and informality 2) ethnicity, origin and nationality 3) age 4) gender and sexualities. Countering Stigma were participation and inclusion, language and labelling, transparency and Accountability
67	Nega Assefa, [28]	Africa	A phone-based survey of 900 HCWs in Burkina Faso, Ethiopia, and Nigeria (300 per country)		Even though only a small proportion of participants reported physical violence and service denial, most perceived social stigma toward HCWs, with 88% of HCPs in Ethiopia reporting social stigma. The majority of the HCPs perceived social stigma due to COVID-19
68	Josephine Enekole Aitafo, [37]	Nigeria	cross-sectional	“220 of HCWs (doctors, nurses, and other allied HCWs) living	34% of respondents stated fear of being stigmatized as reason of self-medication
69	Bhumika Rajendrakumar Patel, [76]	India	cross-sectional	Participants were recruited from a government-designated hospital for COVID-19 in Sola, Ahmedabad	57.47% of respondents experienced high levels of perceived stigma. one of the factors which predict high burnout is high perceived stigma. Nurses had high perceived stigma. Perceived stigma is positively correlated with burnout (*r* = 0.26, *P* < 0.001) with its both components, disengagement (*r* = 0.19, *P* < 0.001) and exhaustion (*r* = 0.30, *P* < 0.001)
70	Jeff Huarcaya-Victoria, [125]	Peru	cross-sectional correlational survey	174 physicians	Those physicians who perceived stigma from their family members obtained higher levels of depressive symptoms; anxiety and distress
71	Chung-Ying Lin, [126]	Taiwan	cross-sectional	500 COVID-19 frontline HCWs in Taiwan	The significantly positive interrelationships between perceived stigma, depression, anxiety, stress, self-stigma, PTSD, insomnia, and fear of COVID-19 found in the pearson correlations signify that as one of these variables increases, the other correlated variable also increases and vice versa
72	Pradeep Rangasamy, [127]	India	A multi-state cross sectional mixed-methods study, with multi-modal aids	218 respondents Health care settings	Nurse practitioners in this survey stated social stigma from neighbors and public as a trigger for perceived stress
73	Carmen H. Logie, [46]	-	Report	-	The stress from COVID-19 stigma may have analogous mental health impacts, including on healthcare providers. persons most impacted by COVID-19 stigma in research and program development (e.g., addressing access barriers posed by COVID-19 care giving and/or healthcare provider roles, quarantine, mental health challenges)
74	Julia Price, [90]	-	review	Frontline HCWs in the era of COVID-19	one of the universal (e.g., self-help) evidence-based supports and interventions by Tier is support for social stigma
75	Fellenza Spahiu, [128]	Kosovo	interpretive phenomenological	nurses of surgical clinics and clinics other than Infectious Diseases Clinic	Surgical nurses were under social stigma
76	Yerina S. Ranjit, [91]	India	cross-sectional	150 HCWs in India	almost 50% experienced discrimination due to their association with COVID-19 patients. this study found that experience of discrimination was associated with perceived courtesy stigma. Two stigma management strategies (reducing offensiveness and passive acceptance) mediated the relationship between perceived courtesy stigma and perceived stress and depression. It is concluded that perhaps due to depletion of cognitive and emotional resources, HCWs engaged more in social support (bonding) and passive stigma acceptance strategies to alleviate the stress associated with providing COVID-19 patient care
77	K. Tari Selçuk, [129]	Turkey	cross-sectional	420 health professionals working in a university hospital serving as a pandemic hospital in a province of Turkey	Social stigma perception was the negative predictor of compassion fatigue, and the positive predictor of burnout, compassion fatigue and intention to leave the profession

## Results

At this stage, the results are summarized and reported to answer the research questions. Of the 77 studies reviewed for the final analysis, 76 were articles and one was a thesis. The design of the studies was as follows: 37 quantitative studies (34 cross-sectional studies and three surveys), 11 qualitative studies, two mixed-method studies, and 12 reviews (one scoping review, three narratives, one umbrella, four systematic studies, one meta-analysis, and one synthesis), three reports, five opinion pieces, seven letters to the editor, and one secondary analysis. Five studies are multi-country, three from Egypt, three from South Korea, seven from India, two from Iran, two from Nigeria, four from the United States, two from Italy, one from Australia, one from Nepal, one from Jordan, two from Saudi Arabia, one from Greece, one from Jakarta, one from Pakistan, two from Ghana, one from Canada, two from Indonesia, one from Sri Lanka, one from Tanzania, three from Turkey, one from Africa, one from Peru, two from Taiwan, and one from Kosovo (Table 2). According to the study results, the reasons for the stigma associated with COVID-19 can vary greatly from fear of the COVID-19 virus [27–31], infecting family members, feeling dirty and negative self-image [32–34] to limited knowledge about the virus [28, 29].

### Manifestation of stigmatization of HCWs

In most of the studies reviewed, HCWs, particularly those directly serving patients, have faced significant stigmatization amid the COVID-19 pandemic (Table 2). This stigmatization encompasses various forms of mistreatment, including bullying, verbal abuse such as ridicule and insults in public settings, physical assaults like spraying bleach or throwing objects, and even attacks on ambulances [35–38]. Additionally, HCWs have encountered harassment and violations of their social and civil rights, such as being denied access to public transportation, rental housing eviction, and denial of services [37, 39–43]. Moreover, they have experienced ingratitude from colleagues, family members, the public, and neighbors [27, 29, 33, 36, 39, 40, 44–60]. Self-stigmatization has also been reported, along with strains in relationships with friends and negative portrayals in the media [29, 33, 38, 40, 47, 48, 52, 58, 60–63]. In general, given the conditions and risks associated with COVID-19, stigmatization has become a widespread and serious phenomenon that requires programs and solutions to improve the health level of society by protecting workers.

### Consequences related to stigma experienced by HCWs can be as follows

Psychological consequences: These people may suffer from stress, anxiety [20, 27, 32, 39, 41, 42, 47, 62–69], grief, depression [32, 50, 54, 60–62, 66–68, 70–73], and suicidal thoughts [54]. Sadness, grief, feelings of guilt [33, 60, 63], hiding the positive result of the COVID-19 test [63], anger, and rage [58, 62], inability to make decisions and not seeking psychotherapy [33, 52, 56], mental health disorders, long-term psychosocial consequences, PTSD symptoms [68, 74] and fear of virus transmission [6, 75, 76] were also among the psychological consequences.

Physical consequences: Sleep disturbances, fatigue [11, 72, 74, 77], exhaustion [32] and a weak immune system [66].

Professional and social consequences: included compassion fatigue, job burnout [11, 74, 77–79], intention to give up work, social isolation [10, 33, 66, 79], loneliness, avoidance of family, humiliation, communication disorders [61], negative effects on work performance and resilience [39, 57, 80, 81], weakening of social cohesion, increase in rumors, loss of social status, deprivation of social and civil rights, such as deprivation of access to public services [39, 57, 80, 81], reduction in social value and social acceptance [6, 10, 36, 39, 41, 66, 82, 83], emotional deprivation [66, 82], a feeling of anxiety when traveling home from the hospital [84], a tendency to self-medicate and refusal to go to the hospital [37], concern about public opinion, concealment of work details [6, 32], and impact on crisis management [32, 41, 84]. It should be noted that one study mentioned that the stigmatization experienced did not affect work performance [85].

Economic consequences: Those affected suffered economic losses because they were expelled from their place of residence and had to give up their job [6, 32].

### Tangible and intangible effects of COVID-19-related stigma on HCWs

The stigma linked to COVID-19 among HCWs has led to the development of negative beliefs, resulting in hesitancy to seek treatment. This reluctance can negatively impact the healthcare profession and society, as affected workers may avoid undergoing diagnostic tests or seeking necessary treatment [33, 37, 86]. Additionally, their families may also face shame and rejection [9, 87], potentially leading to passive acceptance of the stigma [88].

The unseen effects such as loss of societal trust and adverse effects on HCWs, can also result in significant economic losses for society [32, 89].

### Proactive measures to reduce COVID-19-related stigma among HCWs

Considering the mentioned consequences of dealing with the stigmatization of HCWs in the era of COVID-19 and possible epidemics in the future, the solutions used by workers and the things suggested in some studies were psychosocial support from family, colleagues, officials and community [33, 38, 50, 52, 56, 57, 65, 71], providing financial rewards [33, 56], ensuring safety in the workplace, adequate training about COVID-19 for HCWs and better cooperation in the workplace [11, 30, 47, 77, 78], implementation of programs to reduce stigma, including increased public awareness of the nature of the disease, clear dissemination of information about the disease to the public, appropriate community education, public education about disease management, social care and empathy, appropriate representation of health care professionals and efforts to show staff commitment through the media, and clear public health policy announcements [11, 30, 31, 44, 56, 63, 90], appeal to spirituality [41, 60], adoption of measures to improve social motivation of HCWs by the Ministry of Education and Health [31, 56], strict implementation of preventive measures, peer support, proper adherence to personal protection protocol [66, 85, 91], transparency and accountability [44], provision of evidence-based measures to encourage society to reduce offending by workers [92] and lack of awareness of existing conditions by workers [93].

### Research gaps in COVID-19-related stigma among HCWS

There are significant research gaps in understanding the stigma associated with COVID-19 among HCWs, necessitating further investigation to develop validated tools for assessing HCWs’ COVID-19-related stigma. Moreover, it is essential to explore the variations in stigma across different healthcare roles and conduct comprehensive research on the long-term impacts of stigma on HCWs. It is also crucial to examine how stigma manifests in diverse cultural and regional contexts. Additionally, longitudinal studies are vital to track changes in stigma perceptions over time.

## Discussion

This study aimed to investigate the stigmatization of HCWs during the COVID-19 pandemic. Stigmatization of these workers was evident, leading to adverse consequences that affected healthcare staff, their families, and society. It is essential to consider anti-stigmatization measures. However, there are research gaps regarding COVID-19-related stigmatization of HCWs.

The review highlighted that HCWs globally faced significant stigma during the COVID-19 pandemic, manifesting in various forms such as bullying, verbal abuse, physical assaults, and denial of rights and services. This stigmatization also included self-stigmatization, strained relationships, and negative portrayals in the media. SARS pandemic studies supported these findings, emphasizing social stigma experienced by frontline HCWs [94]. Additional research indicated that HCWs encountered verbal and nonverbal violent behaviors aligned with social stigma expressions [95], possibly stemming from society’s aim to protect itself against the stigmatized situation.

Studies suggested that stigma was fueled by fears related to virus transmission and contracting COVID-19, similar to patterns observed during the Ebola epidemic [96]. Individuals might avoid stigmatized individuals due to perceived harm, potentially influenced by a lack of comprehensive understanding of the virus. HCWs themselves faced stigmatization, partly due to negative self-perception and feeling unclean, leading to self-stigmatization behaviors and isolation in society [97]. Their behavior may stem from a desire to avoid being perceived as outsiders, fostering an “us” versus “them” mentality and exacerbating feelings of isolation in society.

Contradictory findings from a census study [97] highlighted that stigmatization stemmed from viewing stigmatized individuals as unhelpful to society. However, the present study attributed stigmatization within society to a lack of knowledge about COVID-19, including transmission, quarantine, and contagiousness duration. Society’s fear and avoidance of HCWs might be rooted in limited understanding of the disease and the nature of the virus.

According to this study, HCWs experiencing stigma during the COVID-19 pandemic are more likely to develop negative beliefs and be reluctant to seek treatment, which has a detrimental impact on healthcare and societal trust. Families may also face shame, perpetuating stigma and causing economic losses. This study revealed instances of HCWs concealing their occupational identity, including hiding a positive COVID-19 test result, aligning with previous research indicating negative emotions as a consequence of stigmatization [95]. Such concealment likely stems from anticipating negative reactions due to previous experiences with societal interactions, family dynamics, or feelings of shame and social alienation.

This experience of stigma can lead to various consequences falling into four main categories: psychological, physical, occupational-social, and economic. Infectious diseases historically associated with stigma, such as plague, yellow fever, and influenza, have demonstrated how this social phenomenon is influenced not only by disease characteristics but also by social and organizational processes, resulting in discrimination, hostility, and social disharmony [74, 98, 99].

Stigma related to emerging infectious diseases can significantly affect the mental and emotional well-being of HCWs, potentially leading to long-term repercussions on their health and quality of life [70, 100, 101]. Stigmatization can result in discrimination-related stress, decreased self-esteem, reduced self-efficacy [101, 102], and even post-traumatic stress disorder (PTSD) among affected individuals [68, 74]. Additionally, physical effects like sleep disturbances, fatigue, exhaustion, and compromised immune function have been observed in stigmatized HCWs. Addressing these challenges and providing support to HCWs during infectious disease outbreaks is crucial.

The stigma surrounding infectious diseases stems from a combination of disease characteristics, societal beliefs, and organizational responses. This stigma can cause significant harm to both individuals and communities that are stigmatized [103]. This phenomenon can result in various occupational-social consequences, such as compassion fatigue, job burnout, reduced satisfaction, desire to leave the profession, social isolation, loneliness, communication breakdowns, negative effects on work performance and resilience, a weakening of social cohesion, and economic losses [6, 32]. Other studies highlighted impacts on social cohesion, including increased rumors [39, 57, 80, 81], loss of social status, deprivation of social rights, concern about public attitudes [6, 10, 36, 39, 41, 66, 82, 83], concealment of work details, decreased social value and acceptance [6, 32], emotional deprivation, fear of returning home from the hospital, self-medication tendencies, reluctance to seek hospital care, crisis management challenges, economic withdrawal from work, and expulsion from residence [32, 41, 84]. Notably, one study found that stigma did not affect the work performance of HCWs [85], while the World Health Organization (WHO) underscores that stigma is a significant driver of discrimination, exclusion, and human rights violations [91].

Some studies have reported various interventions to reduce the negative impact of COVID-19-related stigmatization of HCWs. These measures include providing psychosocial and economic support services, creating better conditions in the work environment, fostering better cooperation, and providing adequate training to enhance safety and health. Additionally, raising public awareness, disseminating information about the disease, and offering appropriate training to address reactions are important steps in combating stigmatization. Due to stigmatization, the media plays a positive role in highlighting employee engagement, implementing measures to boost social motivation, raising awareness, and educating the public on disease management, care, and social empathy. It is also crucial to promote spirituality and adherence to personal protection protocols while maintaining transparency. These efforts align with WHO recommendations to combat stigma, which include disseminating facts, correcting misinformation, dealing with fake stories, and avoiding emphasizing negative aspects and threatening messages about COVID-19. Utilizing official and reliable sources such as the Ministry of Health, WHO, and UNICEF, and choosing words carefully when speaking are essential strategies in addressing stigmatization [91].

Considering that the most frequently experienced consequence of stigmatization is psychological problems, including stress, attention in this area is crucial. A review study [102] investigating the most effective stress reduction techniques for medical staff who come into contact with patients with severe coronaviruses (SARS, MERS, and COVID-19) emphasized that addressing employees’ psychological issues at an organizational level is vital. In addition to ensuring that interventions are appropriate, safe, and aligned with employees’ preferences, remote services such as recorded relaxation packages, recreational activities, and specific stress reduction techniques like mindfulness-based interventions, diaphragmatic breathing, and effective self-efficacy can be beneficial. As communicable diseases pose a constant threat to human society, it is essential to understand how disease-related stigma impacts the outcomes of disease control measures to develop more responsive public health policies during epidemic outbreaks in the future [90]. The results of the reviewed studies indicate that healthcare and nursing staff face a paradoxical and dual situation. While they are praised and applauded as health heroes from balconies [103], they are also avoided due to the perception of working in corona centers, creating a conflicting situation. The likelihood of problems arising in such a stressful environment is doubled.

Significant gaps in understanding the stigma associated with COVID-19 among HCWs are evident so far. These gaps include the need for validated tools to assess stigma and its long-term impacts, longitudinal studies to track changes in stigma perceptions over time, and exploring variations across different healthcare roles in diverse cultural and regional contexts.

### Limitations

The review implemented a systematic and rigorous search strategy to fulfill its objective of consolidating the most recent scientific findings on COVID-19-related stigma among HCWs. It aimed not only to address the current circumstances but also to enhance comprehension of future challenges associated with stigma. However, it is essential to acknowledge the review’s scope limitation, as it solely included studies in English and Persian, potentially excluding relevant research in other languages. Additionally, the review considered both the advantages and drawbacks of Google Scholar as the primary search engine. While Google Scholar enables access to full texts and diverse sources, its dynamic search algorithms and limited journal indexing may pose limitations. To mitigate these constraints, the review diversified its search sources, incorporating other databases to ensure comprehensive coverage.

### Suggestions for future research

Future research on COVID-19-related stigma among HCWs should focus on several key areas. Longitudinal studies are necessary to track the evolution of stigma over time and its lasting impact on HCWs post-pandemic, providing insights into how stigma changes and its long-term effects on mental health and job performance. Research should also explore how COVID-19-related stigma varies across different cultures and healthcare systems, allowing for the development of culturally sensitive interventions. It is crucial to develop and test the effectiveness of various interventions aimed at reducing stigma, including psychological support, educational programs, and policy changes. Further validation and refinement of stigma measurement tools, such as the COVID-19-related stigma scale for HCWs and the COVID-19 Stigma Scale are needed to ensure they are reliable and applicable in diverse contexts. Additionally, examining how stigma affects job performance, job satisfaction, and retention rates among HCWs can help healthcare organizations develop strategies to support their staff better. Finally, exploring the relationship between public perception of HCWs and the stigma they experience, including the influence of media portrayal and public discourse, can provide valuable insights into mitigating stigma.

## Conclusion

The existing literature underscores the significant stigmatization experienced by healthcare professionals amidst the COVID-19 pandemic, primarily stemming from societal fear and limited virus-related knowledge. This stigma often results in the mistreatment of HCWs and the infringement of their social rights, leading to profound psychological repercussions. The enduring impact of this stigma may persist even in the post-COVID era, creating a psychological dilemma for HCWs torn between their professional duties and societal attitudes. Addressing this issue necessitates a comprehensive understanding across various professional and social domains, alongside the formulation and refinement of strategies promoting community awareness, mutual understanding, and empathy. Past epidemic experiences underscore the inadequacy of interventions, warranting the development of more effective stigma prevention and management programs tailored to HCWs. Moreover, existing reviews have neglected the psychological aspects of stigmatization, highlighting the need for qualitative and systematic research to elucidate stigma’s conceptualization and its psychological implications. Through a combination of qualitative and quantitative studies, hypotheses regarding psychological issues related to stigma can be tested, paving the way for targeted interventions and improved strategies to mitigate the psychological impact of stigma.

## Data Availability

Data cannot be shared openly but are available on request from authors.

## Abbreviations

HCWs: Healthcare Workers
COVID-19: Coronavirus Disease of 2019
WHO: World Health Organization
PRISMA: Preferred Reporting Items for Systematic Reviews and Meta-Analyses
PRISMA-ScR: PRISMA Extended Program for Scoping Review
SARS: Severe Acute Respiratory Syndrome
PTSD: Post-Traumatic Stress Disorder
UNICEF: The United Nations International Children's Emergency Fund
MERS: Middle East Respiratory Syndrome
